# FastSkin^®^ Concept: A Novel Treatment for Complex Acute and Chronic Wound Management

**DOI:** 10.3390/jcm12206564

**Published:** 2023-10-16

**Authors:** Pietro G. di Summa, Nicola Di Marzio, Paris Jafari, Marisa E. Jaconi, Dobrila Nesic

**Affiliations:** 1 Department of Plastic and Hand Surgery, University Hospital of Lausanne (CHUV), University of Lausanne (UNIL), 1015 Lausanne, Switzerland; pietro.di-summa@chuv.ch; 2 AO Research Institute Davos, 7270 Davos, Switzerland; nicola.dimarzio@aofoundation.org; 3 Department of Health Sciences, Università del Piemonte Orientale (UPO), 28100 Novara, Italy; 4 Center for Integrative Genomics, University of Lausanne, 1015 Lausanne, Switzerland; paris.jafari@unil.ch; 5 Department of Basic Neurosciences, University of Geneva, 1211 Geneva, Switzerland; marisa.jaconi@unige.ch; 6 Division of Fixed Prosthodontics and Biomaterials, University Clinic of Dental Medicine, University of Geneva, Rue Michel-Servet 1, 1211 Geneva, Switzerland

**Keywords:** skin wound healing, blood clot, tissue micrografts, pig model, full thickness skin defect

## Abstract

Successful treatments for acute and chronic skin wounds remain challenging. The goal of this proof-of-concept study was to assess the technical feasibility and safety of a novel wound treatment solution, FastSkin^®^, in a pig model. FastSkin^®^ was prepared from skin micrografts patterned in blood using acoustic waves. Upon coagulation, the graft was transferred on a silicone sheet and placed on wounds. Six full-thickness wounds were created at the back of two pigs and treated with either FastSkin^®^, split-thickness skin graft (positive control), a gauze coverage (negative control, NC1), or blood patterned without micrografts (negative control, NC2). Silicone sheets were removed after 7, 14, and 21 days. Wound healing was monitored for six weeks and evaluated macroscopically for re-epithelialization and morphometrically for residual wound area and wound contraction. Tissue regeneration was assessed with histology after six weeks. Re-epithelialization was faster in wounds covered with FastSkin^®^ treatments compared to NC2 and in NC2 compared to NC1. Importantly, an enhanced collagen organization was observed in FastSkin^®^ in contrast to NC treatments. In summary, two clinically approved skin wound treatments, namely micrografting and blood clot graft, were successfully merged with sound-induced patterning of micrografts to produce an autologous, simple, and biologically active wound treatment concept.

## 1. Introduction

Understanding the different phases of wound healing is essential for effective wound management. Improving the current standard of care is essential to address the rising incidence caused by an ageing population and the increasing rates of diseases such as diabetes, malnutrition, and oncology treatments.

The wound-healing process is characterized by a complex interplay of cellular events happening in partially overlapping phases [1]. During hemostasis, platelets gather at the injured area and release various clotting factors to initiate the formation of a blood clot. The clot acts as a temporary seal, preventing further bleeding and creating a protective barrier against infections. During the inflammatory phase, blood clots provide a scaffold for the migration of white blood cells and macrophages at the wound site to remove debris and combat any potential infection. In the subsequent proliferative phase, new blood vessels form, supplying oxygen and nutrients. Fibroblasts enter the healing site and create a framework for the new tissue while epithelial cells migrate across the wound surface to gradually close it. The granulation tissue starts to develop, filling the wound and providing a supportive foundation for further healing. During the final remodeling phase, collagen fibers are reorganized to increase the tensile strength of the healed wound. The scar tissue gradually matures to resemble the surrounding healthy skin.

Acute wounds can be caused by trauma, surgical procedures, or accidents, including burns, lacerations, and abrasions. While most acute wounds heal normally, as described above and with an estimated healing time of three to four weeks [2,3], some may present with excessive scarring, long-term adverse effects, and a need for additional treatments [4]. The acute wound becomes chronic when the delicate balance between the inflammatory process necessary for the initial tissue decomposition and subsequent repair is disturbed [5]. Chronic wounds include skin ulcers resulting from compromised blood supply, immune function, metabolic disorders, medication, or previous local tissue injury. They can occur at different time points during the healing process depending on the underlying factors, namely infection, inflammation, and ischemia [6]. Risk factors contributing to the formation of non-healing wounds include diabetes, ischemia, poor circulation, previous radiation therapy, persistent bacterial contamination, chronic inflammation, continued pressure, repeated trauma, neuropathy, difficulty moving, and systemic illnesses often linked with advanced age [7]. Populations with these risk factors will dramatically rise in the next 10 years. For instance, there are already close to 1 billion persons aged over 60, more than 2 billion overweight adults, and over 500 million persons with diabetes [8]. With chronic wounds affecting 1% of the general adult population, 3.6% of people over 65 years old, and more than 5% of people over 80 years old [9], the necessity for better treatment is evident. 

Non-healing wounds becoming ulcers are often difficult to treat and can be recurrent, resulting in high health-related costs and accounting for 2–3% of healthcare budgets in developed countries [5]. They represent a frequent cause of morbidity, with physiological, psychological, social, and economic consequences for the patient, reducing the quality of life. In the U.S., a Medicare study in 2014 showed that 8.2 million patients were diagnosed with at least one type of wound or wound-related infection. The wound care costs for these patients were conservatively estimated at USD 32 billion and Medicare reimbursement per wound averaged from USD 3415 to USD 11,781 [10]. In western Europe, 1% to 2% of the population experience chronic wounds; the NHS spent GBP 2870 per patient on chronic wounds in the United Kingdom in 2012 and 2013 [11,12,13]. 

Split-thickness skin grafting remains the permanent wound coverage and healing method in particular for burn wounds; however, it has a major drawback: harvesting of the healthy skin results in a secondary skin lesion [14,15]. The epidermal and dermal skin substitutes, comprising either cellularized bioengineered skin grafts or acellular dermal regeneration templates, were developed to promote skin regeneration and minimize the formation of severe scars [16,17]. However, these skin matrices are generally associated with skin harvesting and do not eliminate the consequent donor site morbidity. Moreover, sufficient oxygen and blood supply as well as infection prevention are still the critical parameters for a successful graft take [18,19], particularly for chronic wounds. Lastly, a harvested skin graft can be meshed and partially expanded. However, the availability of healthy skin donor sites can be limited for skin injuries involving an extended surface area (e.g., burns). While several studies have addressed the need for engineered pre-vascularized 3D skin substitutes [20,21,22], their clinical translation is still lacking.

Here, we introduce a novel and simple skin wound treatment—FastSkin^®^—which combines two critical skin healing components: blood clot as an autologous matrix and skin tissue micrografts as a source of cells and growth factors, with acoustic patterning of micrografts to foster an efficient healing process. The choice of an autologous blood clot as a wound healing scaffold is based on the confirmed biological activity of this matrix supporting the physiological healing process as well as the availability and ease of sourcing and handling. Indeed, several studies have provided evidence for successful treatment of acute and/or chronic skin ulcers with blood clots [23,24]. Skin micrografting has been employed as a treatment for skin lesions since 1958 [25] and was subsequently successfully used for acute and chronic non-healing wounds, post-surgical dehiscence, [26,27], post-traumatic wounds, vascular or diabetic ulcers and burns [26,27,28,29,30,31,32,33,34,35]. Clearly, re-engineering the hierarchical complexity of living tissues is a key challenge in regenerative medicine; methodologies able to spatially organize biologically active components within three-dimensional extracellular matrix-like substrates are of paramount importance [36]. Specifically, sound-induced manipulation with low-frequency acoustic waves represents a promising new method to precisely distribute cells, spheroids, organoids, and microtissues [37].

The aim of this preclinical proof-of-concept investigation is to demonstrate the FastSkin^®^ concept applicability in a pig model with respect to its technical feasibility and safety.

## 2. Materials and Methods

### 2.1. Study Design

**Figure 1:**
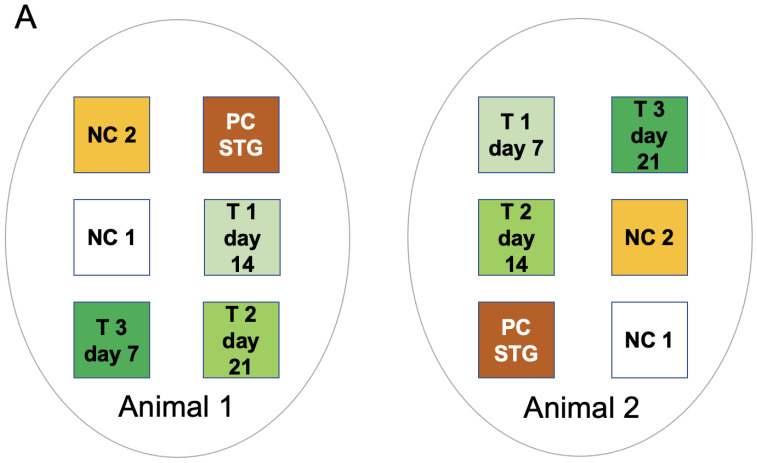

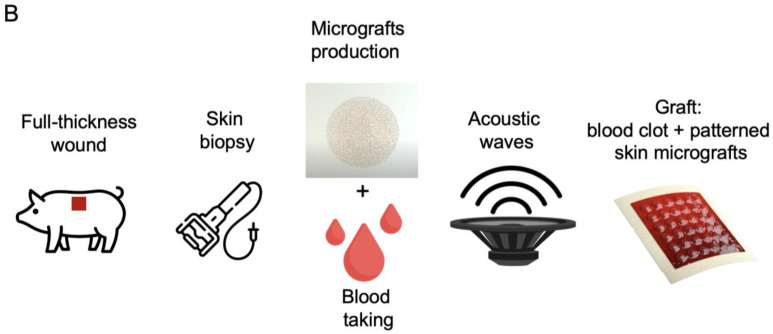
Design and workflow of the pilot study in a pig model. (**A**) The layout of the six wounds created on the back of the two animals is shown. Negative Control 1 (NC 1): only gauze with silicon protective sheet removal after 7 days; Negative Control 2 (NC 2): patterned blood clot without skin micrografts with silicon protective sheet removal after 7 days; Positive Control: MESH split-thickness skin graft (PC STG) with silicon protective sheet removal after 7 days; T1: sound patterned blood clot with skin micrografts (FastSkin^®^) with silicon protective sheet removal after 7 days; T2: FastSkin^®^ with silicon protective sheet removal after 14 days; T3: FastSkin^®^ with silicon protective sheet removal after 21 days. (**B**) Illustration of the experimental workflow.

The study was conducted at the NAMSA facility (Lyon, France; www.namsa.com) according to ISO 10993-6:2016 Biological Evaluation of Medical Devices, Part 6: Tests for Local Effects after Implantation. The ARRIVE (Animal Research: Reporting of In Vivo Experiments) guidelines were followed based on criteria applicable to a pilot study. The NAMSA Ethical Committee approved the protocol of this study under the authorization number APAFIS#23070-2019111311173309 v2. Two female pigs *(Sus scrofa domesticus)* weighing 67 kg at the time of surgery were housed under conditions that conformed to European requirements (Directive EU/2010/63). Six full-thickness skin wounds were created on the back of each pig (three wounds per side). A test (T), positive control (PC STG (split-thickness graft)), and two negative control (NC) treatments were applied (Figure 1A). The test treatment comprised skin micrografts patterned in blood via acoustic waves which, upon coagulation, were mounted on a silicon protective sheet (Figure 1B).

Three FastSkin^®^ treatment conditions were evaluated based on the different time points for the removal of protective silicon sheets: at 7 days (T1), 14 days (T2), or 21 days (T3). The positive control treatment consisted of a MESH split-thickness skin graft (PC STG). Two negative control treatments comprised non-woven gauze (NC1) and blood exposed to acoustic waves without skin micrografts (NC2). 

### 2.2. Animal Care and Surgeries

Animals were allowed two weeks of acclimatization prior to surgeries. Pre-medication consisted of intramuscular injection of tiletamine-zolazepam (Zoletil^®^100, Virbac, Carroz, France), xylazine (Rompun^®^ 2%, Bayer Healthcare, Leverkusen, Germany) and buprenorphine (Vétergesic^®^ multidose 0.3 mg/mL, Ceva Santé Animale, Libourne, France). Each pig was intubated, mechanically ventilated, and placed on isoflurane inhalant anaesthetic (IsoFlo^®^ 100%, Zoetis, Malakoff, France) for continuous general anaesthesia. All wounds were 4.2 cm × 4.2 cm full-thickness skin wounds (until the deep fascia, approximately 800 μm deep) and were created with a scalpel. The protective dressing consisted of non-woven gauze, Tegaderm^®^ (3M, Cergy-ontoise, France) or Opsite^TM^ (Puras AG, Bern, Switzerland), a tubular stockinette, and an elastic adhesive bandage. Paraffin gauze was applied on the top of the wound after the removal of the silicon sheet. An analgesic (buprenorphine, Vetergesic^®^ multidose 0.3 mg/mL, Ceva Santé Animale, Libourne, France) was injected intramuscularly at the end of the surgery day and twice daily for two subsequent days. Animals were observed daily for general health, mortality, morbidity, and the status of the protective dressing integrity. A detailed clinical examination of the animals was performed once a week. The dressings were changed under anaesthesia twice weekly until week 3 and then once a week until study termination. The animals were euthanized by an intravenous injection of a lethal solution (Tanax® (T61: embutramide, mebenzonium iodide and tetracaine hydrochloride), MSD Animal Health, Rahway, NJ, USA). The repaired tissue filling the initial skin wounds was excised to include approximately 1 cm of the skin surrounding the wound site and the first layer of the subjacent muscle and fixed in 10% formalin. 

### 2.3. Preparation of Test and Control Treatments 

**Figure 2:**
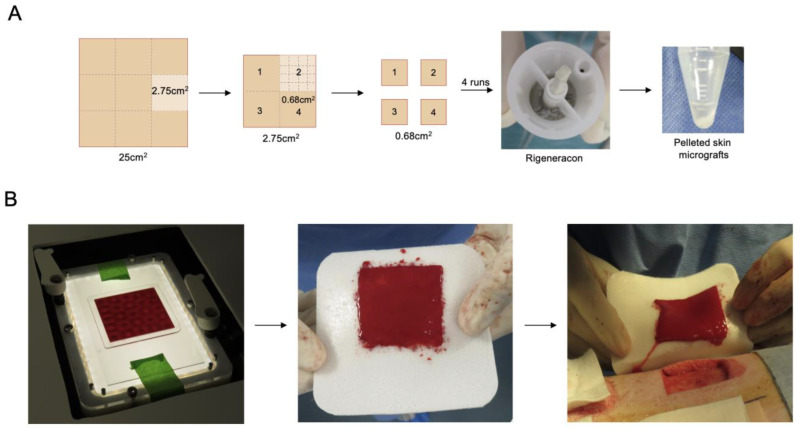
The production of the FastSkin^®^ graft. (**A**) Schematic illustration of the skin biopsy cutting and micrograft preparation process. (**B**) The application of standing sound waves on the blood containing micrografts, transfer of the coagulated blood clot containing micrografts onto the protective silicone sheet, and transfer of FastSkin^®^ graft onto the wound on the pig back.

A 500 µm thick skin layer of 5 cm × 5 cm area was removed from the pig back with Dermatome (Zimmer-Biomet, Zug, Switzerland) using a 5.1 cm blade. The full-thickness wounds of 4.2 cm × 4.2 cm and 800 μm depth were subsequently obtained with a scalpel. A 5 cm × 5 cm and 500 µm thick skin piece was cut with a scalpel into 9 pieces of 2.75 cm² (1.67 cm × 1.67 cm) (Figure 2A). The remaining pieces were kept moist in PBS in a sterile glass container. Each 2.75 cm^2^ piece was next cut into four pieces using a sterile plastic sheet as a template. Each piece was further cut into smaller pieces, collected into 4 mL of PBS, and placed in the Rigeneracon (Rigenera, Human Brain Wave Srl, Turin, Italy) under the blade. The device is a sterile capsule characterized by a grid with hexagonal steel blades (Figure 2A). The rotation at 80 rpm was achieved by a small engine. Rigeneracon was placed in the Rigenera device (Rigenera SICURDRILL 2.0) for a two-minute run. The process was repeated with the remaining three pieces. Two 2.75 cm^2^ pieces were combined per one FastSkin^®^ treatment preparation. All micrografts collected in PBS were transferred in a 15 mL Falcon tube and centrifuged for 5 min at 400 RCF. 

The supernatant was removed and the pelleted micrografts were resuspended in 3 mL of venous blood collected from the jugular vein. Meanwhile, a sterile container was placed onto the sound pattering device ready to receive the blood–micrograft mix. The container was made by binding a laser-cut PMMA frame (60 mm × 60 mm × 1 mm external dimensions, 50 mm × 50 mm × 1 mm internal dimensions) onto a glass slide (70 mm × 120 mm × 1 mm) with a medical grade double side adhesive (3M Science. Applied to Life.^TM^, Bienne, St. Somerville, MA, USA) followed by ethylene oxide sterilization. A total of 2.5 mL of blood with resuspended micrografts was dispensed in the container and vibrated at 60 Hz for 2~3 min using a prototype of the acoustic wave generating device (cymatiX, mimiX Biotherapeutics, Biel, Switzerland). Faraday standing waves were generated at the fluid–air interface aiming for even distribution of the micrografts within the blood volume (Figure 2B). After 2 min, the container was carefully removed from the platform and coagulation was monitored for 20 min. Upon completion of the coagulation, the blood clot with patterned micrografts was transferred on a protective silicone sheet and placed on a wound according to allocation. 

The MESH split-thickness skin graft as the positive control treatment was obtained by removing a skin layer of 5 cm × 5 cm and 500 µm thickness with the Dermatome, manually creating slits with the blade, stretching the skin layer 1.2 times and fixing beyond the wound edges with staples. The negative control represented wounds only covered with non-woven gauze (NC1). The second negative control treatment was prepared in the same way as treatment samples but without skin micrografts (NC2). Positive and both negative controls were covered with the silicone sheets. The placement of treatments and controls was varied on the animals to avoid potential position confounding factors.

### 2.4. Macroscopic Wound Analysis

Wounds were evaluated for the presence of protective dressing, edema, erythema, presence of fluid, signs of infection or maceration, color, granulation tissue, and re-epithelialization. The initial edges of the wounds (visible by the presence of a scar) were marked with a sterile marker for morphometric evaluation of the wound contraction. Quantitative morphometrical analysis was performed on images taken together with a ruler at each observation time point using the morphometry software (Perfect Image^®^ 7.5, Clara Vision, Bièvres, France). The evolution of the non-epithelialized wound surfaces was analyzed and expressed as a percentage of the surface not covered with epithelium. The residual wound surface was expressed as a percentage of the initial wound surface.

### 2.5. Histology

Each excised skin sample was cut into three pieces, fixed in 10% formalin, dehydrated in increasing ethanol concentrations, and embedded in paraffin. A central transversal 4 mm thick section was cut in a latero-medial axis and stained with Hematoxylin–Eosin–SafraninO (HES). All slides were digitalized using a Zeiss AXIOSCAN Zl scanner (Carl Zeiss, Jena, Germany). The qualitative histopathological assessment comprised evaluation of the local tissue effect, including an inflammatory reaction, the presence and appearance of granulation tissue, and re-epithelialization. Semi-quantitative analysis for the presence of collagen bundles within the granulation tissue was also performed employing the following grading: 0 = none; 1 = minimal; 2 = mild; 3 = moderate; and 4 = marked.

## 3. Results

### 3.1. Animal Recovery

**Figure 3:**
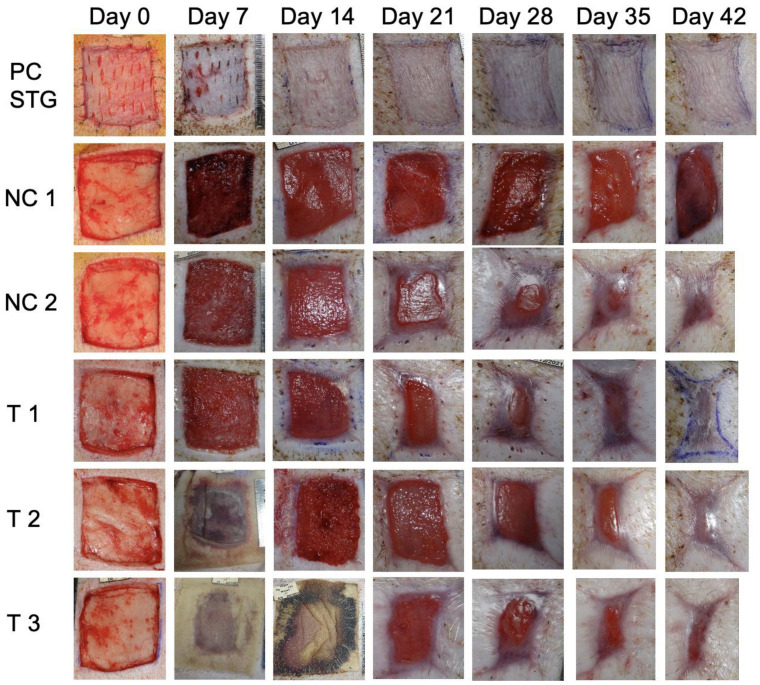
Macroscopic images of wound healing over time. The macroscopic appearance of the wounds from animal 2 is depicted for days 0, 7, 14, 21, 28, 35, and 42. NC 1: only gauze; NC 2: blood exposed to acoustic waves without skin micrografts, PC: split-thickness skin grafts (STG); T1: FastSkin^®^ with protection removal after 7 days; T2: FastSkin^®^ with protection removal after 14 days; T3: FastSkin^®^ with protection removal after 18 days.

The study proceeded without significant events. The FastSkin^®^ protective silicon sheet was removed as planned on day 7 (T1; animals 1 and 2), day 14 (T2; animal 2), and day 18 instead of day 21 (T3; animal 2). The silicon protection sheets slipped from two wounds on day 11 (T2 and T3; animal 1), preventing the planned evaluation with silicon removal on days 14 and 21. For the positive and both of the negative controls, the silicon sheet was removed on day 7. The appearance of wounds over time from animal 2 is presented in Figure 3. Slight signs of inflammation and maceration were observed for a negative control wound (NC 1, animal 2) even 42 days after wound creation. Overall, a slight to moderate oedema was observed for almost all wounds.

### 3.2. Wound Healing

For FastSkin^®^-treated wounds, the first granulation tissue was observed on approximately day 7 (Figure 3), reaching completion between days 11 and 18. The first signs of re-epithelialization were observed on day 11 in all uncovered wounds, and re-epithelialization was complete on day 35 for wounds T1 and T2 animal 1 and T2 animal 2, and day 42 for T2 animal 2, and T3 animals 1 and 2. For the split-thickness skin graft (STG) positive controls, one wound was considered completely healed on day 11 and the other on day 28. For NC 1, the first granulation tissue was observed on day 7 and was complete on day 18. The first signs of re-epithelialization were observed on day 11, with the complete re-epithelialization on day 42 for one wound (animal 1). The second wound was not healed even after 42 days (animal 2). For NC 2, the first granulation tissue was observed on day 7 and was complete on day 18. The first signs of re-epithelialization were seen on day 11, with the complete re-epithelialization on day 35 for one wound (animal 1) and day 42 for the other wound (animal 2). The data indicate faster re-epithelialization for the wounds covered with the patterned blood clot (NC 2) compared to gauze only (NC 1) as well as between any FastSkin^®^ treatment (T1-T3) and NC 2. 

### 3.3. Morphometric Analysis of Wounds over Time

**Figure 4:**
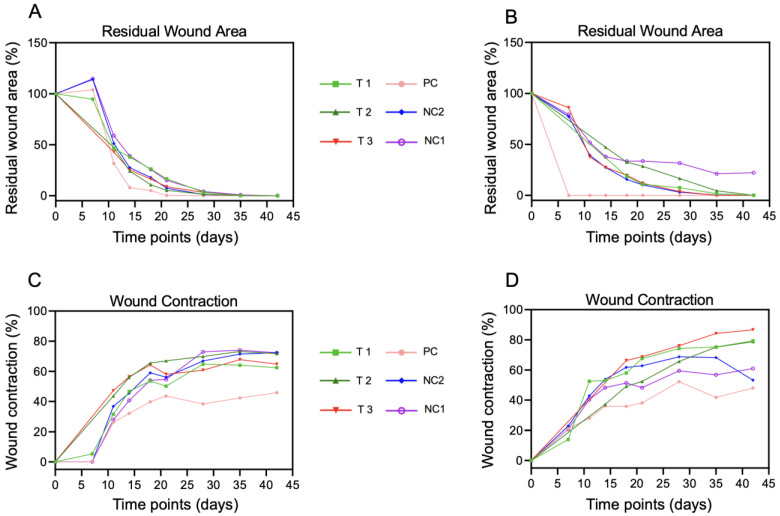
Quantitative morphometric analysis of wound healing over time. (**A**) The percentage of residual wound surface ((**A**): animal 1 and (**B**): animal 2) and wound contraction ((**C**): animal 1 and (**D**): animal 2) were measured at indicated intervals until day 42.

All treated wounds demonstrated wound closure in three phases: slower between day 0 and day 7, accelerated from day 7 to day 14, and slower from day 14 to day 42 (Figure 4A). The fastest wound closure occurred in the split-thickness graft-treated wound after 7 and 21 days. Only one wound treated with non-woven gauze (NC 1 animal 2) did not close after 42 days. 

For almost all wounds, a high wound contraction was observed on day 11, followed by linear contraction (Figure 4B). Three wounds treated with FastSkin^®^ showed approximately 80% wound contraction 42 days after wound creation. By contrast, two positive control wounds showed a reduced wound contraction (approximately 50%) at the end of the study.

### 3.4. Histology Evaluation

Overall, all wound sites comprised a thick layer of granulation tissue at the dermis level covered by a well-differentiated epidermis. The granulation tissue showed evidence of maturity, i.e., the presence of blood vessels, fibroblasts, and an abundant collagenous matrix. 

**Figure 5:**
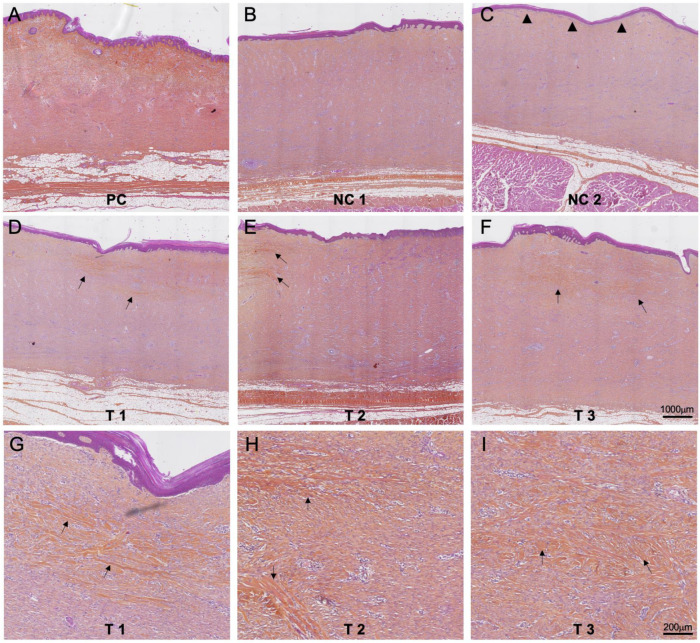
Histology evaluation of skin repair. Hematoxylin–Eosin–SafraninO staining of histologic sections from the middle of the treated wounds from animal 2 (except NC 1) collected at day 42. PC: positive control, NC 1: negative control 1; NC 2: negative control 2. Formation of epidermis characteristic rete-pegs is absent in wounds treated with blood clots without micrografts (indicated by arrowheads). Arrows indicate the appearance of collagen bundles in all FastSkin^®^ treated samples. (**G**–**I**) panels are magnified images (**D**–**F**), illustrating the formation of collagen bundles.

In the positive control sites (PC), the subepidermal granulation tissue showed abundant collagen fibers (stained orange), giving these areas a normal dermal appearance, except for the absence of adnexal structures, with minimal inflammatory reaction (Figure 5A). The negative control 1 demonstrated features described above for one wound (animal 1, Figure 5B) while the other wound (animal 2) did not show signs of re-epithelialization (the wound which did not close after 42 days). In the negative control 2, the dermis had an appearance similar to the test and negative control 1 group. However, the epidermal basal membrane was linear, i.e., without signs of reticulation (Figure 5C). All wounds treated with FastSkin^®^ test treatment showed similar dermis and epidermis features as described above, regardless of the time of removal of the protective silicon sheet. In contrast to both negative controls, an onset of collagen bundle formation was visible (Figure 5D–F), accompanied by slight inflammatory local tissue effects. A semiquantitative assessment of collagen bundles’ presence within the wound granulation tissue confirmed the presence of collagen bundles in FastSkin^®^-treated wounds. An overview of all findings is presented in Table 1.

## 4. Discussion

Successful treatments for acute and chronic wounds remain elusive. In this study, we introduced FastSkin^®^, a new treatment concept combining two well-established wound healing strategies, namely skin micrografts and clotting blood, with a new technology for micrograft sound patterning. 

We performed a preliminary safety and technical feasibility proof-of-concept study in a pig model of a full-thickness skin wound. The healing of the wounds treated with FastSkin^®^, a split-thickness skin graft (positive control), an inert coverage dressing (gauze, negative control NC 1), or patterned blood without micrografts (negative control NC 2) was monitored for six weeks. The wound coverage with a split-thickness skin graft yielded the fastest and most efficient healing. In clinics, however, this type of treatment generates new wound sites that can lead to complications, particularly in patients with poor wound healing capacity, such as the elderly, diabetics, or oncology patients. Moreover, the autologous donor skin surface necessary for treatment can be quite limited in patients with large wounds such as burns. In this preclinical pilot study, the treatment with biologically active components, i.e., a patterned blood clot alone or FastSkin^®^, suggests faster wound healing. Further studies are, however, required to validate these observations. Removal of the protective dressing at different time points (7, 11, 14, or 18 days) within the limitation of the study, i.e., low number of samples, had no effect on residual wound coverage or wound contraction. The structure of the epidermis differed between a patterned blood clot and FastSkin^®^ treated wounds, with rete ridges absent in wounds treated without micrografts. However, the major difference distinguishing the applied treatments was the quality of the skin tissue at complete healing. While all the wounds were closed (except for one NC1), only wounds treated with FastSkin^®^ showed visible formation of collagen bundles in the dermis at the histology level. More abundant collagen bundles in the dermis observed in FastSkin^®^-treated samples suggests better quality of wound healing. Importantly, FastSkin^®^ treatment demonstrated the possibility of reducing the size of the donor site by extending the biopsy by a factor of 4.5, leading to decreased post-operating complications and a favorable aesthetic outcome. Finally, skin collection for FastSkin^®^ preparation could be achieved by tissue harvesting at multiple sites (mosaic harvesting), a technique that could minimize the above-mentioned donor site complications.

The micrograft-based strategy has emerged as a new, effective, and affordable approach for wound treatment. Notably, skin tissue micrografts represent a living ecosystem with all the cells of interest—namely epithelial cells, fibroblasts, and melanocytes—which reside within a native extracellular matrix and can boost healing within the wound bed. Among the mechanisms evoked in micrograft-based wound healing, an improvement of skin re-epithelialization [38] was shown via acceleration of fibroblast and keratinocyte migration in an ERK signaling-dependent manner [39]. Faster wound healing may be influenced by granulation tissue formation, MMP activity, organized collagen content, and newly formed blood vessels. Interestingly, micrografts in mice were shown to induce a transcriptional signature that is enriched in genes related to wound-healing-associated biological processes. Those are likely mediated through micrografts’ release of multiple growth factors such as VEGF, HGF, G-CSF, PDGFs, and TGFα [39,40]. Particularly, HGF was shown to stimulate motility and the endothelial-to-mesenchymal transition in keratinocytes in 3D to become mesenchymal cells that contribute to tissue regeneration [40].

Recently, two clinical micrografting approaches were developed: the Xpansion Micrografting System (Applied Tissue Technologies LLC, Hingham MA, USA) with a 100-fold expansion efficiency [38] and the “Rigenera micrografting technology” [26,27,28,31,33]. By employing both technologies, the obtained micrograft-containing solution can be either directly deposited onto the wound or combined with scaffolds (such as collagen sponge) or biomaterials to form a bio-complex. The final biological cocktail contains micrografts with all the necessary cell types and tissue-specific extracellular matrix promoting correct cell interactions with their physical environment as well as corresponding tissue-specific growth factors for appropriate signaling and morphogenetic processes for wound healing [41]. 

Upon skin wound bed preparation for graft placement, pronounced bleeding is essential for successful graft adherence and acceptance upon transplantation. Blood favors the distribution of nutrients and oxygen, waste removal, the regulation of pH levels, and prevention of infections. Furthermore, it supplies red and white blood cells, platelets, proteins, clotting factors, minerals, electrolytes, and dissolved gasses. In a tightly orchestrated time and space process, the forming blood clot develops into a protective fibrin-based scaffold matrix for cell repopulation, thereby promoting wound healing. Topically applied, autologous blood clots stimulate angiogenesis and lower bacterial bioburdens via immune cells. Autologous blood clots hence represent an ideal biological delivery system allowing the timely release of cytokines and growth factors [42].

The essential advantages of the autologous blood clot are easy access, preparation, and immediate application at the point-of-care. An ideal wound treatment’s key characteristics include optimal adherence, physiological water vapor transport, elasticity yet mechanical stability, a bacterial barrier, absence of toxicity and antigenicity, hemostatic activity, ease of application and removal, minimal storage requirements, and low costs [24]. Thus, from the regulatory perspective, blood clots meet the requirements of living biologics composed of a metabolically active matrix with viable autologous cells. Recent studies have provided evidence for successful treatment of acute and/or chronic skin ulcers with blood clots [23,24]. Blood clot-based products are already in clinical use. The ActiGraft^®^ system marketed by RedDress Medical (Ponte Vedra Beach, FL, USA) was recently successfully applied on hard-to-heal wounds [43]. The proven clinical benefits of a blood clot in wound healing, in addition to easy access, preparation, and immediate application at the point-of-care, were the central rationale for the selection of blood clot as the delivery matrix for the FastSkin^®^ concept. Furthermore, this novel approach alleviates another challenge in applying micrografts or even cells, namely the homogenous distribution of biological actors within the wound bed. 

The process of morphogenesis, occurring during development and which is dysregulated in many pathologies, is based on the self-patterning of cells [37,44,45]. Acoustic or sound-induced manipulation to remotely control the spatial cellular organization within a carrier matrix has arisen as a promising method to fabricate cell-laden constructs [46,47,48,49]. This technology takes advantage of hydrodynamic forces exerted by standing waves on particles, cells, spheroids, organoids, or microtissues within a liquid medium to create distinct patterns in response to the applied frequency and amplitude. To date, acoustic manipulation has advanced from micro- or nanoparticle arrangement in 2D to the assembly of multiple cell types or organoids into highly complex in vitro 3D tissues [37]. Low-frequency acoustic vibrations (10–200 Hz) offer a cell-friendly environment in which standing waves condensate cells without affecting their integrity, cytoplasm, or phenotype [50,51,52]. Since the applied frequency domain is sensibly below the ultrasound regime (kHz–MHz), heating and cavitation effects cannot occur. Moreover, the cells experience the drag forces only for a short time (1–2 min), further hindering any negative impact on cell viability and metabolism [53]. This technology was successfully applied to generate blood vessel constructs in vitro and in a mouse ischemia model by combining human adipose stem cells and endothelial cells with catechol-conjugated hyaluronic acid hydrogels [48]. Additionally, the acoustic patterning of myoblasts in gelatine–methacryloyl hydrogels significantly enhanced myofibrillogenesis and promoted the formation of muscle fibers containing aligned bundles of myotubes [46]. Recently, sound induced manipulation (SIM), exploited in the FastSkin^®^ concept, was successfully used to generate a multiscale organized vascular network combining human bone marrow mesenchymal stem cells (MSC) and endothelial cells with GelMA and fibrin gel [52] as well as a drug testing platform based upon the angiogenic response of tumor spheroids [54]. 

**Figure 6:**
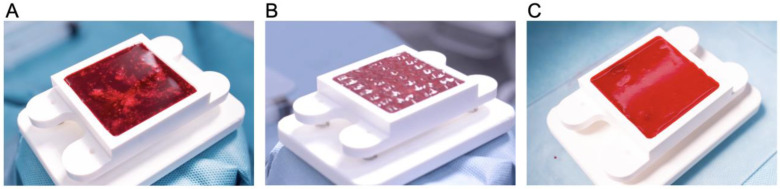
Patterning of skin micrografts in blood by acoustic waves. (**A**) Skin micrografts obtained from pig skin were minced in human blood. (**B**) Static sound waves were applied to distribute the micrografts. (**C**) Upon 20 min, completion of blood coagulation was observed, thereby fixing skin micrografts in the desired patterned position.

Acoustic manipulation (or SIM), adopted in the presented FastSkin^®^ treatment, leverages the blood as a liquid phase for the standing wave formation and the tissue micrografts as bioactive tissue particles. To the best of our knowledge, the application of sound patterning to manipulate homologous and biologically active materials for the development of an implantable construct has not been previously documented. The individualization and spatial distribution of skin micrografts are achieved through low-frequency static waves (in the range of 10 to 100 Hz) generated with an intro-operative sound centrifuge within a container (sound cartridge) (Figure 6). Given that this study represents a pioneering proof-of-concept for the implementation of acoustic manipulation in an intra-operative context, several unique challenges need to be addressed.

In this context, we present the workflow underlying the FastSkin^®^ treatment, which has demonstrated its successful application in an intra-operative setting. Notably, there was no evidence of infection or impaired healing at the wound sites following the processing of autologous materials. Our primary objective was to achieve a homogeneous distribution of standing waves at the fluid–air interface to enhance the dispersion of micrografts across the entire surface of the container, thereby minimizing areas with limited micrograft presence and consequently restricting therapeutic potential. 

Current limitations of the FastSkin^®^ approach reside in the difficulty of observing the precise location of tissue micrografts within the blood without additional processing. This challenge arises from the non-transparent nature of liquid blood. Further investigations will be required to elucidate the localization of micrografts within the blood clot. Moreover, we envision characterizing the freshly prepared FastSkin^®^ treatment through histological cross-sections to assess their exact distribution within the construct. Our preliminary data indicate that tissue micrografts can be manipulated using sound patterning and effectively observed in a saline solution. These results are a prerequisite for future preclinical efficacy studies with an appropriate number of animals and more detailed analyses of healed skin structure and function, in particular the quantification and/or visualization of specific markers for epidermal differentiation, barrier formation, or extracellular matrix composition.

## 5. Conclusions

**Figure 7:**
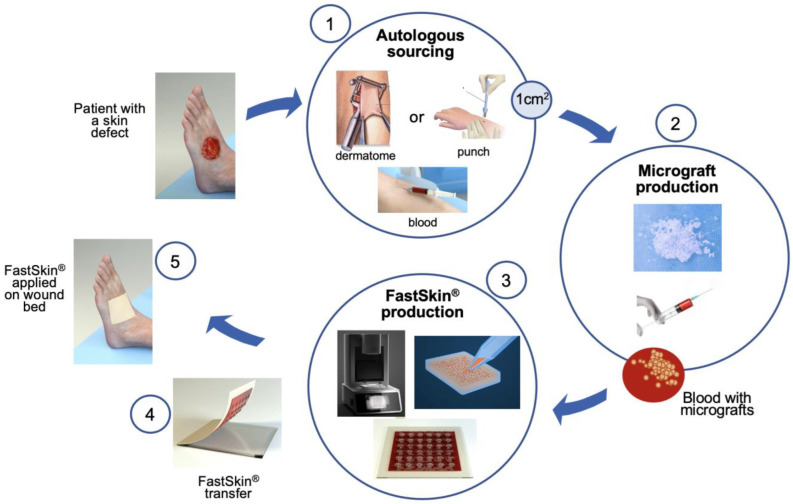
FastSkin^®^ workflow at the point-of-care. The five steps are depicted: (1) autologous skin biopsy harvesting with either dermatome or biopsy punch and blood withdrawal from the vein, (2) skin micrograft production, (3) application of acoustic waves for micrograft patterning in blood, (4) transfer of coagulated blood clot with micrografts to the wound protection matrix, and (5) the application of FastSkin^®^ onto the patient’s skin wound.

Overall, two clinically approved techniques in skin wound treatment, namely micrografting and blood clot graft, were successfully merged with acoustic patterning of micrografts to produce FastSkin^®^, an autologous, simple, and biologically active wound treatment concept. The FastSkin^®^ concept can be easily prepared by the clinical staff in the operating room or outpatient care facilities from available autologous primary material with no major technical hurdles. The proposed fabrication process is shown in Figure 7. The FastSkin^®^ concept can be topically applied for the management of different types of cutaneous wounds, including leg ulcers, pressure ulcers, diabetic ulcers, burns, and mechanically- or surgically-debrided wounds.

This proof-of-concept safety and technical feasibility study in a pig model of a full-thickness skin wound demonstrated that FastSkin^®^ can be prepared as a biological graft in a timely manner and easily applied on the wound bed. Moreover, FastSkin^®^ is also proved to be safe and suggests a better dermis tissue quality than gauze or patterned blood clots without micrografts. The future studies will address the current limitations associated with the precise localization of micrografts following acoustic manipulation. Additionally, we intend to conduct a comparative analysis of their therapeutic efficacy in the FastSkin^®^ treatment against non-acoustically manipulated counterparts.

## Figures and Tables

**Table 1:** Summary of the data. * designates the wound that did not close.

	Silicone Removal (Day)	Wound Contraction (% of Initial Wound) D42	Residual Wound Surface (% of Initial Wound) D28	Histology Score
PC	7	46	0	4
PC	7	48	0	4
NC 1	7	72	4	0
NC 1	7	61	32	0 *
NC 2	7	72	1	0
NC 2	7	53	3	0
T1	7	62	3	1
T1	7	79	4	2
T2	11	72	1	1
T2	14	79	17	1
T3	11	65	3	1
T3	18	87	7	2

## Data Availability

Supporting data can be obtained from the corresponding author upon reasonable request.
